# Implantable cardiac monitors in cryptogenic stoke: Clarity or added uncertainty?

**DOI:** 10.1016/j.hroo.2022.04.002

**Published:** 2022-04-18

**Authors:** Anand Thiyagarajah, Edmund Cheong, Dennis H. Lau

**Affiliations:** ∗ Department of Cardiology, Royal Adelaide Hospital, Adelaide, Australia; † Centre for Heart Rhythm Disorders, The University of Adelaide, Adelaide, Australia; ‡ Department of Neurology, Royal Adelaide Hospital, Adelaide, Australia

Although up to one-third of ischemic strokes do not have a clearly defined mechanism and are labeled as *“cryptogenic,”* many affected individuals are suspected of having subclinical atrial fibrillation (AF). Defining the stroke etiology directly impacts management. Most patients are treated with antiplatelet therapy alone in the absence of AF because empirical initiation of anticoagulant therapy has not been shown to improve outcomes in several studies.^1^^,^^2^ The impact of AF detection and consequent gain from anticoagulation therapy formed the premise for several randomized control trials that investigated prolonged rhythm monitoring strategies in patients with cryptogenic stroke.^3^^,^^4^ Although these studies confirmed that event-triggered recorders and implantable cardiac monitors (ICMs) provide a much higher yield for AF detection, they did not assess the impact of early AF detection on hard endpoints such as recurrent stroke and mortality. A study to clarify this unaddressed gap in the knowledge has been long overdue.

In this issue of *Heart Rhythm O*^*2*^*,* Yaghi et al^5^ provide a real-world perspective on how the intensity of cardiac rhythm monitoring can impact outcomes in cryptogenic stroke. Using data derived from a United States claims database, the authors compared longitudinal outcomes in patients who received ICMs to those who received external cardiac monitors (ECMs) only. This retrospective claims-based analysis included data from 12,994 patients with mean follow-up of 3.17 years for ECM patients and 1.92 years for ICM patients. The rates of AF detection were high in both arms (49% and 40%) compared to the CRYSTAL AF (Cryptogenic Stroke and Underlying Atrial Fibrillation) study (30% at 3 years), although the cohort in the present study was older (∼67 vs ∼62 years) and consisted of more female participants (∼54% vs ∼36%), despite lower mean CHA_2_DS_2_-VASc score (∼2.6 vs ∼3.0).^4^^,^^6^ In an analysis adjusted for baseline clinical characteristics, the key finding was that the use of ICMs was associated with reduced time to AF detection, reduced time to initiation of oral anticoagulation, and lower all-cause mortality. The results highlight the need to be vigilant for AF in cryptogenic stroke and provide a persuasive argument to further investigate the merits of early ICM implantation.

The past decade has seen tremendous advancements in ICM technology, including improved electrogram acquisition, AF detection algorithms, and remote monitoring capabilities.^7^^,^^8^ Despite these advancements and the cumbersome, inconvenient nature of ambulatory monitoring, ICMs have not been routinely adopted in the workup of cryptogenic stroke. Even in the present study by Yaghi et al,^5^ only 10% of included patients received an ICM. Although others have suggested ICMs are a cost-effective alternative to ambulatory monitoring, harnessing the additional information they provide to improve patient outcomes remains a challenge.^9^ The risk of stroke in individuals with AF is a complex, time-dependent interaction between rhythm, atrial contractile function, and comorbidities that promote a prothrombotic milieu. Although there are robust data confirming that subclinical AF carries an increased risk of thromboembolism, quantifying this risk has proved difficult, and this complicates decisions surrounding anticoagulation.^10^

First, the burden of subclinical AF that constitutes an elevated stroke risk remains elusive. Studies involving nonstroke populations with implanted pacemakers or defibrillators suggested that, beyond a certain threshold, a dose–response relationship between subclinical AF burden and stroke risk exists. However, the minimum burden of AF that defines an elevated risk remains unclear.^11^^,^^12^ Second, the temporal proximity of an AF episode to a stroke event that implies causation has not been established. In TRENDS (Prospective Study of the Clinical Significance of Atrial Arrhythmias Detected by Implanted Device Diagnostics), 45% of patients with device-detected AF who suffered cerebrovascular events or systemic emboli had no atrial arrhythmia in the 30 days before their event.^12^ In some patients, AF may simply be a manifestation of an elevated cardiovascular risk profile rather than the direct mechanism of their stroke. Therefore, whether a brief episode of device-detected AF several months after a stroke merits anticoagulation is unknown. Third, risk factors independent of AF status may contribute to occult cardioembolism in cryptogenic stroke. In the present study, the mortality benefit seen in the ICM arm may have been due to early anticoagulation in patients without AF. Approximately 20% of patients in both arms received anticoagulation despite never developing AF, and patients who received an ICM seemed to receive anticoagulation much earlier. In fact, although the overall time to anticoagulation was 57% faster in patients receiving an ICM, this value fell to only 12% when the analysis was restricted to patients diagnosed with AF. This result suggests that variables other than AF detection may have influenced anticoagulation in the ICM arm as well as any associated mortality benefit. It also is plausible that the ability of novel oral anticoagulation therapy to inhibit atrial fibrosis and development of the AF substrate might have contributed in part.^13^ For example, there is an evolving concept of an atrial myopathy whereby myocardial fibrosis and a stiff, poorly contractile left atrium can lead to increased blood stasis. Identifying atrial myopathy in the clinical setting through biomarkers, advanced electrocardiographic signal processing, or magnetic resonance imaging may further assist risk stratification of AF risk in those with cryptogenic stroke, to guide patient selection for ICM use and maximize its cost-effectiveness, and warrants further investigation.^14^

In summary, this thought-provoking study by Yaghi et al^5^ raises several important issues. Although we cannot confidently infer a causative link between earlier AF detection and reduced mortality, the results emphasize the need for randomized control trials to confirm such a relationship. Consideration also must be given to the practicalities of ICM use, including streamlining referrals between neurologists and implanting cardiologists, managing false-positive alerts that frequently occur with remote monitoring, and expediting oral anticoagulation if a critical burden of AF is exceeded.^15^ Despite the complexities surrounding anticoagulation therapy, it is evident that subclinical AF is common in cryptogenic stroke, and clinicians must recognize associated cardiometabolic risk factors and treat them aggressively. At the same time, we are reminded that many patients with cryptogenic stroke do not manifest AF, and equal attention should be paid to other stroke etiologies such as unstable plaque due to symptomatic nonstenotic carotid disease, with further research needed to delineate potential cardioembolism due to atrial myopathy without AF. Meanwhile, as we seek to clarify the importance of subclinical AF and better define the role of ICMs and oral anticoagulation therapy in cryptogenic stroke, clinicians must remain on the lookout for clinical AF in which data supporting anticoagulation are unequivocal.
